# Plain Language Summary

**DOI:** 10.2903/j.efsa.2023.p210902

**Published:** 2023-09-14

## Abstract

This publication is linked to the following EFSA Journal article: https://efsa.onlinelibrary.wiley.com/doi/10.2903/j.efsa.2023.8213

efsa JOURNAL

14 September 2023

## Scientific and technical assistance on some aspects related to housing and health of cats and dogs in commercial breeding establishments

### Overview of the EFSA scientific report

- This scientific report provides technical assistance to the European Commission (EC) for preparing policy options for the legal framework of commercial breeding of cats and dogs. The report focuses on the welfare of cats and dogs bred commercially for sport, hunting and companion purposes.
- The welfare aspects assessed concern: the type of housing and the housing conditions, the health of queens (unspayed female cats) and bitches in relation to breeding requirements and the painful procedures in place for convenience surgery.
- The European Food Safety Authority (EFSA) provided, where possible, the scientific evidence to support the recommendations included in the 'Guidelines for the responsible breeding of cats and dogs' produced by a Voluntary Initiative of the EU Platform on Animal Welfare.

### Why is EFSA writing about this topic?

- The protection of cats and dogs in commercial facilities has not yet been regulated in detail in the EU's animal welfare legislation. Legislative proposals are foreseen on the protection of cats and dogs when kept as companion animals and for sport in commercial breeding.
- The EC requested EFSA to assess the recommendations provided by the EU Platform on Animal Welfare for the preparation of policy options for the legal framework of commercial breeding of cats and dogs.

### What was EFSA asked to do?

- The EC requested EFSA to review the available and relevant scientific literature and judge if there is scientific evidence to support the recommendations from the EU Platform on Animal Welfare on three main animal welfare aspects for cats and dogs in the context of commercial breeding: housing, health and painful procedures.

### How did EFSA carry out this work and what data were used?

- The main source of evidence was the published scientific literature. This was reviewed to see if sufficient evidence exists to support the recommendations on the three welfare aspects.
- The literature was complemented by ad hoc consultation with experts. Where possible, examples of national legislation, implemented by EU Members states and other countries, were considered.

### What were the limitations/uncertainties?

- The main sources of uncertainty were listed in the scientific report and were mainly related to the available scientific evidence. The evidence used was obtained from research articles and reviews. Further evidence might exist outside of traditional academic publications (the 'grey literature').
- An important limitation was the time constraint which did not allow a full risk assessment that would normally consider a multidisciplinary approach with different stakeholders, different experts and a thorough peer review process.

### What were the outcomes and their implications?

Given these limitations/uncertainties, the evidence found by EFSA supports the following recommendations.

- **In relation to type of housing and exercise**
  - Cats and dogs should not be kept permanently in boxes, cages or crates.
  - Breeding dogs should have an area, preferably outdoors, in which to socialise and exercise regularly, but it is unclear if this should be daily.

- **In relation to housing temperature**
  - To prevent thermal stress, cats should be housed at a temperature between 15°C and 26°C. However, the range may be wider for certain breeds and type of cats.
  - To prevent thermal stress in dogs, insufficient evidence could be found to support the temperature range of 10-26°C, as high variation is observed across different breeds and types.
  - Kittens kept without their mother during the first 3 weeks of life, and puppies kept without their mother during the first 10 days of life, should be kept at a higher temperature than when kept with their mother.
  - Factors such as breed, size, coat, fur and level of activity affect how dogs and cats can regulate their temperature. These should be taken into account to define the optimal temperature range for keeping them.

- **In relation to housing light**
  - Light affects the biology of animals. The importance of setting specifications for artificial light for cat welfare is supported by scientific evidence. For breeding cats and dogs, a period of darkness every day is recommended to maintain the circadian rhythm.
  - Evidence to support the need for *natural* daylight in breeding cats and dogs was not found.

- **In relation to health**:
  - Queens and bitches should not be bred before skeletal maturity (before they are fully grown). Skeletal maturity is breed specific and should be checked before breeding.
  - To prevent exhaustion, the physical state of the queens should be checked before breeding through the assessment of their general health status and body condition.
  - Queens older than 6 years and bitches older than 8 years should be checked by a veterinarian before being used as breeding animals.
  - Concerning the minimum breeding age for dogs, the size of the animal is relevant. Although there is no scientific evidence in favour of a general minimum breeding age of 18 months, for small breeds 18 months is suggested as they will be fully grown by that age. Larger breeds should reach skeletal maturity before breeding.
  - Bitches should preferentially be bred from the second oestrus onwards.

- **In relation to painful surgical interventions:**
  - Convenience or cosmetic surgery, such as declawing in cats and ear cropping, tail docking, and vocal cord resections ('debarking') in dogs, should not be performed unless necessary for the health of the animals.

### What are the key recommendations for further research?

The scientific report recommended further research in the following areas.

- **In relation to housing**
  - The need for access to natural daylight and the impact on cats' and dogs' welfare.
  - The need for daily access to an outdoor area (dogs).
  - Defining the optimal temperature (thermal neutral zone) for specific breeds of cats and dogs.
  - The optimal temperature conditions for keeping kittens and puppies during the first 3 weeks of life and 10 days of life respectively.
  - Identifying animal-based measures (e.g. shivering, curling up, heart rate) for assessing thermal stress (heat and cold stress) and monitoring animal welfare.
  - The optimal dark and light duration as well as parameters for artificial light (i.e. intensity, spectrum and duration).
- **In relation to health**
  - The minimum period between kittenings (cats giving birth) and whelpings (dogs giving birth) as well as a minimum breeding age for queens and for large dogs.

**Disclaimer**

- This plain language summary (PLS) is a simplified communication of EFSA's *Protection of cats and dogs in commercial breeding*.
- The full EFSA scientific report can be found here: https://doi.org/10.2903/j.efsa.2023.8213.
- The purpose of the PLS is to enhance transparency and inform interested parties on EFSA's work on the topic using simplified language to present a summary of the main findings.
